# Toxic Anterior Segment Syndrome with Intracameral Moxifloxacin: Case Report and Review of the Literature

**DOI:** 10.1155/2021/5526097

**Published:** 2021-03-02

**Authors:** Annahita Amireskandari, Andrew Bean, Thomas Mauger

**Affiliations:** Department of Ophthalmology and Visual Sciences, West Virginia University, Morgantown, WV, USA

## Abstract

A case of severe anterior segment toxicity secondary to high-volume, undiluted intracameral moxifloxacin for endophthalmitis prophylaxis is reported. We examine the other reported cases of toxicity after intracameral moxifloxacin, as well as iris depigmentation and transillumination syndromes after oral and topical fluoroquinolone exposure. Additionally, we review the literature on safety, efficacy, and appropriate dosing of intracameral antibiotics with a focus on moxifloxacin.

## 1. Introduction

Toxic anterior segment syndrome (TASS), though rare, is one of the most dreaded complications of anterior segment surgery. Resulting in often significant anterior segment inflammation, corneal edema, and damage to the iris and angle structures, it has been reported after cataract surgery, penetrating keratoplasty, and intravitreal antivascular endothelial growth factor (VEGF) injections and vitreoretinal surgery. TASS is thought to be the result of toxicity from residue on surgical instruments, disinfectants, medication, and/or preservatives in medications used during surgery [1]. However, the causative agent remains unknown in many cases.

Moxifloxacin is a fourth-generation fluoroquinolone with broad spectrum activity against Gram-positive and Gram-negative bacteria [2]. Vigamox (Alcon, Fort Worth, Texas) is commonly used in the US as an off-label intracameral injection during cataract surgery, as it is preservative free. We present a case of TASS associated with intracameral preservative-free 0.5% moxifloxacin (Vigamox) during otherwise uncomplicated cataract surgery.

## 2. Case Report

A 74-year-old female presented as a referral from an outside ophthalmologist for persistent corneal edema and mydriatic pupil in the left eye after cataract surgery. According to the referring surgeon, the patient underwent routine phacoemulsification and insertion of a single-piece acrylic intraocular lens implant (IOL) (Acrysof UltraSert ACU0T0, Alcon, Fort Worth, TX). Approximately 0.2 ml intracameral preservative-free 1% lidocaine was instilled in the beginning of the case. Intracameral preservative-free moxifloxacin (Vigamox) was used to replace the anterior chamber at the end of the case (a total volume of approximately approximately 0.6 cc). The surgeon was inadvertently given 0.5% undiluted Vigamox in the syringe instead of the ordered 0.1% concentration for all 7 of his cases that day. This error went unnoticed until the following day when all 7 patients had more than the anticipated amount of intraocular inflammation. Though we do not have details regarding the other 6 cases, this patient was noted to have significant corneal edema, elevated intraocular pressure (IOP), and anterior segment inflammation. These signs persisted at her postoperative week one visit, raising concern for TASS. She was initially treated with topical prednisolone acetate, Vigamox, ketorolac, timolol, netarsudil (Rhopressa 0.02%, Aerie Pharmaceuticals, Irvine, CA), and hypertonic saline (Muro 128 0.5%, Bausch + Lomb, Rochester, NY). The anterior chamber inflammation resolved after several weeks, but the corneal edema and fixed, dilated pupil with transillumination defects persisted at 2 months postoperatively. Per the referring provider, she was the only patient of the 7 that day to have persistent corneal edema and iris damage requiring further surgical intervention. To our knowledge, no cultures or PCR was performed postoperatively. Of note, the patient had cataract surgery in the right eye 2 months prior with the same type of IOL (Alcon Acrysof UltraSert ACU0T0) and 0.6 cc of 0.1% intracameral Vigamox without complication.

**Figure 1:**
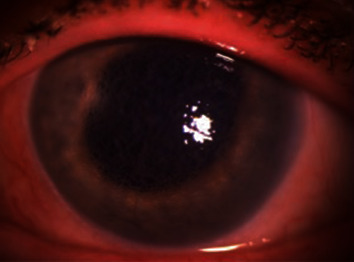
Slit lamp photograph of the left eye with diffuse light.

The patient presented to our institution approximately two months after cataract surgery in the left eye. The uncorrected vision was 20/25 in the right eye and count fingers at 3 feet in the left eye. The left pupil was dilated and slightly irregular with minimal to no reaction to light. The IOP was 15 mmHg in the right eye and 21 mmHg in the left eye. The anterior and posterior segment exams were normal with a centered posterior chamber IOL on the right. The left eye had mild ptosis with mild conjunctival injection. The cornea was noted to have limbus-to-limbus bullous keratopathy. The anterior chamber was deep without frank inflammation. The iris was fixed, dilated and slightly irregular with a large temporal transillumination defect. It was difficult to assess for endothelial pigment deposition given the diffuse corneal bullae. The posterior chamber IOL appeared to be well centered in the capsular bag (see Figure 1). A hazy view posteriorly revealed no obvious abnormalities of the fundus.

It was evident that there was significant corneal endothelial damage, which led to the chronic bullous keratopathy and toxic injury to the iris, resulting in an atonic iris with an irregular pupil in the left eye. After discussion with the patient, the decision was made to proceed with endothelial keratoplasty and iris repair.

**Figure 2:**
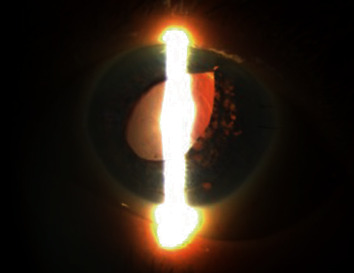
Slit lamp photograph with retroillumination of the left eye one month postoperatively.

**Figure 3:**
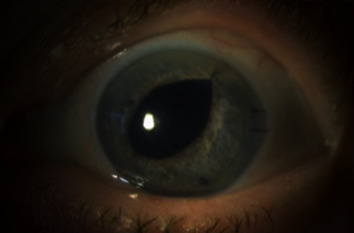
Slit lamp photograph of the left eye with diffuse light one month-postoperatively.

The patient did well postoperatively with a well-adhered graft at her postoperative day one visit and clear cornea at her postoperative week one visit. At week one, the IOP was elevated to 28 mmHg so brimonidine was started twice daily (BID). The pressure fluctuated from 19-26 mmHg over the next 3 months so timolol BID was added as well. Prednisolone acetate was tapered slowly and eventually switched to fluorometholone daily. Visual acuity was 20/40-2 in the left eye at postoperative week one and month one visits. She had 2 nylon sutures in the main wound. Figures 2 and 3 were taken at her one-month postoperative visit. At her most recent follow up 9 months after surgery, she remained on fluorometholone daily, timolol BID, and brimonidine BID. Her best corrected visual acuity was 20/25, and IOP was 18 mmHg in the left eye.

## 3. Discussion

The etiology of toxic anterior segment syndrome in this case is thought to be due to instillation of a high volume (approximately 0.6 cc) of undiluted 0.5% intracameral Vigamox. However, other causes of intraocular inflammation should always be considered, especially infectious etiologies. We are not aware that any cultures or PCR was performed for this patient. Additionally, one must also consider contamination or alteration of the intracameral balanced salt solution, lidocaine, and viscoelastics, as well as, detergents or residue on surgical instruments. Given that all cases that day had an unexpected amount of postoperative inflammation and the Vigamox concentration was the only known deviation from standard procedure at that particular surgical center, this was thought to be the most likely cause of TASS.

In August 2020, the FDA released a report citing 29 cases of TASS associated with intracameral moxifloxacin use up through December 19, 2019 [3]. Sixteen of these cases involved compounded drugs using moxifloxacin as a bulk substance; 10 involved repackaged Moxeza (Alcon, Fort Worth, Texas) moxifloxacin. There were three cases in which it was unknown whether the moxifloxacin had been repackaged or diluted; two of these were Vigamox, and one was Moxeza. It is important to note that both Vigamox and Moxeza are FDA approved for topical use only [3]. Additionally, Moxeza contains xantham gum which has been associated with TASS [1, 3].

Details of several cases of toxicity with intracameral moxifloxacin have been published this year [4–7]. All 4 of the cases in the series by Sanchez-Sanchez et al. occurred after glaucoma surgery in which patients received Vigamox brand moxifloxacin intracamerally and subconjunctival mitomycin C [4]. Light and Falkenberry presented a case that occurred after pars plana vitrectomy in which the only intraocular medication administered was Vigamox. Regarding the two cases associated with cataract surgery, one involved Vigamox brand moxifloxacin [6]. In the other case by Peñaranda-Henao et al., it was unknown what dose or brand of moxifloxacin was used as the surgery had been performed at an outside institution [7]. It is also unknown whether any other intraocular medications were administered in either of these cases, but 1% preservative-free lidocaine is commonly given intracamerally during cataract surgery. Similar to our patient, all of these cases involved pigment dispersion in the anterior segment. The presence of anterior segment inflammation, pupillary abnormalities, and elevated IOP varied between reports. The individual cases described in these studies are summarized in Table 1.

Multiple reports of uveitis, bilateral acute iris depigmentation (BADI), and/or transillumination (BATI or BAIT) syndromes with *oral* fluoroquinolones use have also been published. These syndromes often include intraocular inflammation, diffuse pigment dispersion onto the corneal endothelium and trabecular meshwork, elevated IOP, pupillary sphincter damage, and iris atony. It is unclear whether the elevated IOP seen in many of these cases is due solely to pigment clogging the meshwork or whether there is direct medication toxicity to the trabecular meshwork tissue [8–24]. Of particular interest, a retrospective analysis of 22 cases of acute iris transillumination by Kawali et al. identified 17 cases in which topical ophthalmic fluoroquinolones were used either alone or in conjunction with an oral fluoroquinolone [12].

Opinions regarding the risks and benefits of intracameral moxifloxacin vary greatly. Multiple studies have reported decreased risk of postoperative endophthalmitis with intracameral moxifloxacin use [25–31], though it may be argued that there is more data regarding the use of intracameral cefuroxime [32–35]. While intracameral cefuroxime has been studied more extensively than moxifloxacin and is more cost effective, moxifloxacin may have other benefits. Several authors cite that dose-dependent killing may be an advantage of moxifloxacin over cefuroxime and vancomycin [26, 34]. In addition to their group's clinical experience with moxifloxacin and vancomycin, Arshinoff et al. published an extensive literature review of intracameral vancomycin, cefuroxime, and moxifloxacin during cataract surgery. They concluded that intracameral moxifloxacin is more effective at preventing endophthalmitis compared to vancomycin and cefuroxime, citing that bacterial resistance to moxifloxacin is overcome at a safe level within the anterior chamber [26].

The most effective choice of prophylactic antibiotic remains unclear in the current literature. Bowen et al. performed a meta-analysis of the safety and efficacy of intracameral cefuroxime, moxifloxacin, and vancomycin. The authors note that both cefuroxime and moxifloxacin can be used to decrease the risk of postoperative endophthalmitis safely [27]. Another in vitro study of bacteria incubated on IOLs showed that all three antibiotics were effective against streptococcus and propionibacteria. They also found that moxifloxacin had broader coverage than cefuroxime and vancomycin, despite being less effective against staphylococcus and pseudomonas at lower doses [36]. Most authors agree that, while retinal toxicity is rare with vancomycin, it should be avoided for routine endophthalmitis prophylaxis during cataract surgery [26, 27, 34].

Regarding volume and concentration of intracameral moxifloxacin, there is some disagreement in the literature about what provides the safest and most efficacious endophthalmitis risk reduction. Several groups have reported favorable safety and efficacy results with undiluted 0.5% moxifloxacin at small doses of 0.03 ml [30] and 0.1 ml [31]. However, Shorstein and Gardner observed that smaller injection volumes of higher concentration moxifloxacin resulted in less precision in the delivered dose. Compared with a 0.5%/0.1 ml intracameral injection, flushing the anterior chamber with 0.15%/0.5 ml provided similar residence times but more consistent anterior chamber concentrations [37]. Matsuura and colleagues examined the safety and efficacy of total replacement of the anterior chamber with 50-500 mcg/ml of moxifloxacin in over 18,000 cataract surgery cases and noted a decreased risk of endophthalmitis. Additionally, there was no significant endothelial cell loss or cases of TASS [38]. They found similar results when flushing that anterior chamber and bag [39]. Arshinoff et al. also found a decreased risk of endophthalmitis with minimal risk of adverse events with 0.3 to 0.4 cc of diluted (3.0 cc Vigamox with 7.0 cc balanced salt solution) moxifloxacin for a final dose equal to 450-699 mcg [25]. Arbisser compared 0.1% moxifloxacin to cataract surgery without intracameral antibiotics and found no significant adverse events with moxifloxacin administration [40].

Both animal and human studies have provided conflicting results regarding moxifloxacin's effects on anterior segment structures. Akal et al. used a rat model to evaluate the effects of intracameral moxifloxacin and noted higher oxidative stress parameters and apoptotic activity in the corneal tissues of rats receiving moxifloxacin compared to controls [41]. Conversely, another study on rabbit eyes found no significant toxicity to endothelial cells with intracameral cefazolin, levofloxacin, or moxifloxacin compared to controls [42].

Haruki et al. used cultured human endothelial cells to examine the effects of different concentrations of moxifloxacin, levofloxacin, and cefuroxime. They found that moxifloxacin doses of more than 500 mcg/ml caused damage to cell membranes and decreased cell viability. Thus, they recommended using an intracameral dose of 500 mcg/ml or less. Another group studied the effects of moxifloxacin on human endothelium, trabecular meshwork, and retinal pigment epithelial cells and found no toxicity to any of these structures with concentrations up to 150 mcg/ml. The authors argued that, given moxifloxacin's minimum inhibitory concentration to inhibit 90% of the most common pathogens causing postoperative endophthalmitis (MIC 90) [43], a concentration of 150 mcg/ml should be safe and effective at preventing endophthalmitis [44]. A very recent in vivo study compared intracameral moxifloxacin doses of 250 mcg/0.1 ml and 500 mcg/0.1 ml during cataract surgery and found no significant difference in endothelial cell count postoperatively. The authors state that a higher concentration should be considered to decrease the risk of endophthalmitis, as both concentrations appeared safe [45]. These results should be interpreted with caution, however, as toxicity from fluoroquinolones may depend on exposure time [46].

The use of intracameral antibiotics still varies greatly among surgeons. As reviewed here, there is conflicting data on the risks and benefits of intracameral moxifloxacin. Regardless of antibiotic choice, it is critical that the specific drug and concentration are checked at each step of preparation. Depending on the surgical center or hospital's protocol for preparing antibiotics, this may involve a hospital or facility pharmacy, operating room nurses, scrub technicians, and/or physicians. By verifying the antibiotic name, whether or not it is preservative free, its concentration, and the planned injection amount at each step of preparation, critical errors are less likely to occur. The surgeon is ultimately the last check in this process and should also verify each of these parameters prior to instilling any medication in the eye. If an error does occur and results in significant anterior segment toxicity as seen with the case presented, initial aggressive control of inflammation and IOP is indicated. Even with a severe inflammatory response, it is often possible to have relatively good outcomes with proper management.

## Figures and Tables

**Table 1:** Case reports of toxicity associated with intracameral moxifloxacin.

Publication	Subject	Indication for intracameral moxifloxacin	Moxifloxacin brand	Moxifloxacin dose	Other intra-op medications	Adverse effects (AEs)	Time to onset of AEs	Postoperative medications started prior to onset of AEs
Light and Falkenberry 2019	1 of 1	Endophthalmitis prophylaxis for PPV for symptomatic floaters	Vigamox	0.1 ml of 5 mg/ml	None	Decreased VA, AC flare, pigment dispersion in AC and angle, iris depigmentation, iris TID, elevated IOP	Post-op week 3	Unspecified topical steroid, NSAID, fluoroquinolone
Peñaranda-Henao et al. 2020	1 of 1	Endophthalmitis prophylaxis for cataract surgery	Unknown	Unknown	Unknown	Ocular pain, headache, elevated IOP, AC inflammation with keratic precipitates, pigment dispersion in the AC and angle, pupil deformation with poor reactivity, iris atrophy and TID, pigmented cells in the anterior vitreous, mild decrease in foveal brightness	Post-op week 2.5	Oral acyclovir, intramuscular betamethasone, topical prednisolone acetate, topical timolol. Later, acyclovir was switched to valacyclovir
Sanchez-Sanchez et al. 2020	1 of 4	Endophthalmitis prophylaxis for express shunt implantation for POAG	Vigamox	0.1 ml of 5 mg/ml	Subconjunctival mitomycin C 0.2 mg/ml	Pigment dispersion in the AC, filtering bleb, and angle around the express shunt, mild mydriasis with sluggish light reaction	Post-op week 4	Topical moxifloxacin, diclofenac sodium, predinoslone acetate
Sanchez-Sanchez et al. 2020	2 of 4	Endophthalmitis prophylaxis for express shunt implantation for POAG	Vigamox	0.1 ml of 5 mg/ml	Subconjunctival mitomycin C 0.2 mg/ml	Pigment dispersion in the AC and filtering bleb, fixed middilated pupil	Post-op week 2	Topical moxifloxacin, diclofenac sodium, predinoslone acetate
Sanchez-Sanchez et al. 2020	3 of 4	Endophthalmitis prophylaxis for deep non-penetrating sclerotomies with supraciliary hema implant for glaucoma	Vigamox	0.1 ml of 5 mg/ml	Subconjunctival mitomycin C 0.2 mg/ml	Pigment dispersion in AC and on endothelium, mydriasis with poor light reaction, iris TID, elevated IOP at post op month 3 secondary to pigment obstruction of the trabeculo-Descemet membrane	Post-op week 4	Topical moxifloxacin, diclofenac sodium, predinoslone acetate
Sanchez-Sanchez et al. 2020	4 of 4	Endophthalmitis prophylaxis for deep non-penetrating sclerotomies with supraciliary hema implant for POAG	Vigamox	0.1 ml of 5 mg/ml	Subconjunctival mitomycin C 0.2 mg/ml	Pigment dispersion under bleb, TID, irregular pupil	Post-op week 6	Topical moxifloxacin, diclofenac sodium, predinoslone acetate
Zubicoa et al. 2020	1 of 1	Endophthalmitis prophylaxis for cataract surgery	Vigamox	0.1 ml of 5 mg/ml	Unknown	Eye pain, circumciliary congestion, pigment deposition on the IOL and in the angle, flare, mydriasis, iris TID	Post-op week 3.5	Unspecified topical steroid

AC: anterior chamber; AEs: adverse effects; IOP: intraocular pressure; NSAID: nonsteroidal anti-inflammatory; PPV: pars plana vitrectomy; POAG: primary open angle glaucoma; TID: transillumination defect; VA: visual acuity.

## Data Availability

The data used to support the findings of this study are included within the article.
